# An Overview of Recent Development in Composite Catalysts from Porous Materials for Various Reactions and Processes

**DOI:** 10.3390/ijms11052152

**Published:** 2010-05-18

**Authors:** Zaiku Xie, Zhicheng Liu, Yangdong Wang, Qihua Yang, Longya Xu, Weiping Ding

**Affiliations:** 1 Shanghai Research Institute of Petrochemical Technology, Sinopec, 1658 PuDong Beilu, Shanghai, 201208, China; E-Mails: lotuslzc@yahoo.com (Z.L.); wangyd.sshy@sinopec.com (Y.W.); 2 State Key Laboratory of Catalysis, Dalian Institute of Chemical Physics, Chinese Academy of Sciences, 457 Zhongshan Road, Dalian 116023, China; E-Mails: yangqh@dicp.ac.cn (Q.Y.); lyxu@dicp.ac.cn (L.X.); 3 Lab of Mesoscopic Chemistry, Department of Chemistry, Nanjing University, Hankou Road 22, Nanjing 210093, China; E-Mail: dingwp@nju.edu.cn

**Keywords:** composite catalysts, porous materials, catalysis, combination

## Abstract

Catalysts are important to the chemical industry and environmental remediation due to their effective conversion of one chemical into another. Among them, composite catalysts have attracted continuous attention during the past decades. Nowadays, composite catalysts are being used more and more to meet the practical catalytic performance requirements in the chemical industry of high activity, high selectivity and good stability. In this paper, we reviewed our recent work on development of composite catalysts, mainly focusing on the composite catalysts obtained from porous materials such as zeolites, mesoporous materials, carbon nanotubes (CNT), *etc*. Six types of porous composite catalysts are discussed, including amorphous oxide modified zeolite composite catalysts, zeolite composites prepared by co-crystallization or overgrowth, hierarchical porous catalysts, host-guest porous composites, inorganic and organic mesoporous composite catalysts, and polymer/CNT composite catalysts.

## 1. Introduction

In the refining and chemical industry, most processes (above 80%) involve catalysis and catalysts. Catalysts are indispensable to many environmental procedures from automotive exhaust treatment to industrial effluent and municipal waste treatment, which contribute to reducing pollution and other adverse environmental impacts. Besides, catalysts are also key technologies in developing fuel cells and photovoltaic cells to replace conventional fossil fuels. Since most of the practical catalytic processes are so complicated that single component catalysts cannot easily meet the requirements for high performance such as high activity, remarkable selectivity and good resistance to deactivation, composite catalysts, which have two or more components combined together, are much more researched and used. According to the combination method of the components, there are several kinds of composite catalysts, as listed below:

- Simple physical mixtures [1–4].
- Crystallite composites obtained by intergrowth, overgrowth/core-shell growth or co-crystallization [5–8].
- Composites with active components and/or promoters coated (attached, doped, loaded, supported or deposited) on the support [9–12].
- *In-situ* combinations prepared by sol-gel hydrolysis or co-precipitation followed by calcination [13–16]
- Matrix materials modified with metal oxides, metal hydroxides, amorphous SiO_2_, organic motifs, sulfonic acid groups, *etc.* [17–20].
- Host-guest composites, which are prepared by incorporation of guest compounds into host materials [21–24].
- Hybrid composites, which comprise a matrix material (polymer, carbon, *etc.*) in which one or more material phases are dispersed, including polymer/carbon nanotube composites, PVC/nano-SiO_2_ composites, PVC-ZnO composite films, polypyrrole (PPy)/C composites, *etc.* [25–28].
- Hierarchical porous composites, which have pore systems of micropores, mesopores and macropores combined together [29–31].

Although it is doubtful that every component contributes to the catalytic performance, there must be some effective components that do. The mechanism for such good effects is the key point, and these effects may be one of those listed below:

♦ Simple supporting effect [32,33].
♦ Stabilizing the microstructure or active components [34,35].
♦ Formation of new compounds which act as active components or stabilizers [36,37].
♦ Having two or more functions [38–42].
♦ Dual-function effect in elementary reactions [43].
♦ Adjusting the performance of acid and base sites [44–47].
♦ Controling of surface bonding state, coordination state and electronic state [48–51].
♦ Controling of redox performances [52–55].
♦ Influencing the speed of adsorption/desorption and diffusion of molecules [56–59].
♦ Influencing the migration or transference of the active species such as O, H, *etc.* [60–63].
♦ Incorporating guest active compounds or centers into host materials, which provides heterogeneous catalysts [21–24].
♦ Enhancing the mechanical, thermomechanical, electronic or other physical and chemical properties, especially for polymer matrix composite catalysts [25–28].

Nowadays, processes like light olefin (C_2_^=^ to C_4_^=^) production and the production of aromatics and their derivatives such as aromatic isomerization, disproportionation, and alkylation are of great importance in the petrochemical industry, and great advances have been made in the catalytic procedures including maximum propylene production, methanol to olefins (MTO), methanol to propylene (MTP), toluene selective disproportionation, heavy aromatics disproportionation, aromatics synthesis from LPG, and ethylbenzene and cumene manufacture and other small volume petrochemical manufacture. Besides the catalyst technology for petrochemical industry, chiral catalysts have become another new focus of development in the fine chemical industry. These processes require advanced catalysts and technologies, on which we have put our R&D emphsis.

As we know, porous materials such as zeolites, carbon, mesoporous materials and other molecular sieves are some of the most popular catalytic materials, widely used in the refining and chemical industry. Due to the rapid diminishing of fossil resources and great demand for protecting the environment, it is urgent to develop new catalysts with high performance, particularly with porous materials. Besides, it would be best to design catalysts employing atomic economy principles and apply them to the industry.

Over the recent several years, we placed our efforts on the development of composite catalysts based on porous materials. In this review paper, we will overview some of our achievements on this area. Six types of porous composite catalysts are discussed, including amorphous oxide modified zeolite composite catalysts, zeolite composites obtained by co-crystallization or overgrowth, hierarchical porous catalysts, host-guest porous composites, inorganic and organic mesoporous composite catalysts and polymer/CNT composite catalysts.

## 2. Amorphous Oxide Modified Zeolite Composite Catalysts

### 2.1. Amorphous Silica Decorated Zeolite Composite Catalysts

Toluene disproportionation and transalkylation is an important process that converts low-value toluene and heavy aromatics into high value mixed xylenes. In the traditional toluene disproportionation process, an equilibrium mixture of xylenes is produced after which the *para-*xylene product has to be recovered by either crystallization or selective adsorption, and the remaining xylenes are recycled through the xylene isomerization process. In order to raise *para-*xylene selectivity, a new process known as selective toluene disporportionation has been developed recently which produces *para-*xylene with selectivitity greater than 90%, and the product is obtained directly without needing a subsequent isomerization process.

**Figure 1.**
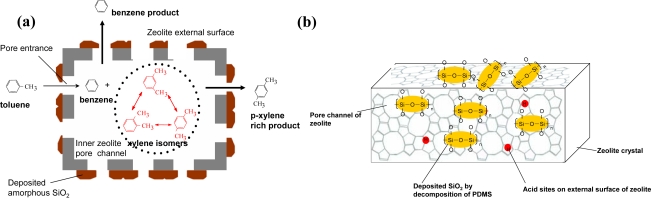
**(a)** Scheme of selective toluene disporportionation reaction on amorphous silica decorated zeolite composite catalysts; **(b)** illustration of the external surface active sites of the zeolite modified by polysiloxane PDMS. Reproduced from Reference [77]. Copyright 2007 Elsevier.

It is known that the inner micropore channels of zeolites have shape-selective properties, while the external surface active sites of the zeolite crystallites are none shape-selective. When toluene molecules enter the pore channels of the zeolite, they disproportionate to benzene and an equilibrium mix of three xylene isomers (see Figure 1a). Because of their greater diffusivity, benzene and *para-*xylene leave the pores more rapidly and the remaining *ortho*- and *meta*-isomers re-equilibrate to form more *para-*xylene (shape-selective property). While on external surface, if there are some acid sites, re-isomerization of the *para-*xylene may occur rapidly, which results in a decrease in the final *para-*xylene selectivity of the catalyst. Thus, in order to improve the *para-*xylene selectivity, it is necessary to deactivate the acidic sites on the external surface of zeolite to suppress xylene isomerization. By modification, in addition to the decrease of the overall number of acidic sites present on the zeolite, the size of the pore entrances on the external surface is also decreased to increase the relative diffusivity of *para-*xylene by several orders of magnitude compared to the larger *ortho*- and *meta*-isomers. Therefore, the *para-*xylene selectivity will increase. However, the activity of the catalyst will be reduced to some extent, so zeolite modification should be implemented carefully to achieve the best balance between zeolite diffusivity and external surface activity.

A variety of modification methods have been adopted to improve the catalytic selectivity of ZSM-5 for toluene disproportionation or alkylation reactions such as impregnation of metallic or non-metallic compounds, chemical vapor deposition of silica (SiO_2_-CVD), pre-coking [64–66] and chemical liquid deposition of silica (SiO_2_-CLD) modification. For the catalysts prepared by these methods, supporting metallic or non-metallic compounds, such as H_3_PO_4_, CaO, MgO and Fe_2_O_3_, on ZSM-5 could eliminate the external acidic sites of zeolite, but they also greatly decrease the acidic sites in channels [67–70]. The on-line pre-coking modification of zeolite is often difficult to carry out, and this modification has to be conducted again after regeneration of zeolite catalyst [71,72], so SiO_2_-CLD modification was adopted instead to prepare a commercial ZSM-5 catalyst for shape-selective disproportionation of toluene to *para*-xylene [73]. It was reported in Mobil’s patents that in methylation of toluene or toluene disproportionation, high selectivity for *para*-xylene (up to 90%) may be reached by adjusting the CVD or CLD conditions and selecting polysilaxone as SiO_2_ precursor [74–76]. CVD is commonly used in the laboratory, however, CLD is more easily transferred to large-scale industrial preparation.

After careful comparison, SiO_2_-CLD modification is regarded as the preferred method for industrial preparation of the catalysts for the selective toluene disporportionation process, and the zeolite composites decorated with amorphous silica are among the most suitable catalysts. Recently, we have made detailed studies on the method [77], only to find that by SiO_2_-CLD modification, polysiloxane is first adsorbed on ZSM-5 via Van der Waals force and hydrogen bond interactions (Figure 1b). The deposition of polysiloxane as silica on ZSM-5 is a combined process of the degradation of the acid-catalyzed cracking with the thermal pyrolysis, followed by oxidation at high temperature. SiO_2_-CLD modification mainly occurs on the external surface of zeolite, and does not destroy the framework structure of ZSM-5. Besides, most of the acidic framework bridging hydroxyls and non-framework hydroxyls remain after SiO_2_-CLD modification, while the silanol hydroxyls of the zeolite almost disappear. On the other hand, the amount of acid of the modified ZSM-5 is less than that of the parent ZSM-5, and it drops gradually when the content of modified SiO_2_ increases, but the acidic strength distribution of ZSM-5 is unchanged during modification. After four-cycle modification, the catalytic activity of external surface is reduced by almost 92%, making the external surface of the modified almost inactive, while the acidity and activity of the inner pores of the zeolite composite catalyst remain at almost the same levels as those of the parent ZSM-5. Therefore, SiO_2_-CLD with polysiloxane is an ideal modification for obtaining the high shape-selective ZSM-5 catalyst.

In 2007, we successfully developed a new amorphous silica decorated zeolite composite catalyst which we called SD-01 [78,79]. This catalyst has been successfully applied in pilot plants by the Tianjing and Yangzi petrochemical corporations. It should be noted that small ZSM-5 crysallite particles (less than 200 nm) are used here for preparation of this catalyst. Although it is more difficult to modify the external surface by polysiloxane on small crysallites than on large ones, this catalyst has lower benzene/xylene ratio (less than 1.4) than the catalysts made from large zeolite crysallites (above 1.4), because it has shorter diffusion path of small crystals and leads to lower decomposition of xylene to benzene.

### 2.2. Phosphorus Oxide Modified Zeolite Composite Catalysts

Propylene is an important basic chemical intermediates which is produced primarily as a byproduct from petroleum refining or from ethylene production by steam cracking of hydrocarbon feedstocks. The petrochemical industry is presently facing a major squeeze in propylene availability as a result of the increasing demand for propylene derivatives (e.g., polypropylene). The traditional methods for propylene production are not satisfactory to meet this increasing demand. Recently, the catalytic cracking of mixed C_4_- or C_5_-olefins for producing propylene and ethylene over various zeolite catalysts, such as ZSM-5 [80–84], ZSM-48 [85], ZSM-23 [85], and MCM-22 [86] has been a research focus. The zeolites were found to have high selectivity and yield of propylene in the C_4_-olefin cracking reaction, but because of the strong polymerization tendency of the olefin-containing reactants and/or products on the acidic catalysts, one of the most critical problems for such reaction is catalyst deactivation. Phosphorus oxide modified zeolite composite catalysts have been researched for dozens of years [88–92], and it has been proved that phosphorus modification would be an effective method to elevate the hydrothermal stability of ZSM-5. The role of phosphorus in the catalytic cracking over ZSM-5 catalyst, however, has seldomly been reported so far. Most studies believe that the hydrothermally stable acid sites of the modified HZSM-5 originate from the framework aluminum being protected from dealumination. The changes in the structure of the modified acid sites during hydrothermal treatment have scarcely been investigated, and few convincing results on the phosphorous status under hydrothermal conditions in the modified HZSM-5 have been elucidated to date. It seems that the details of the interaction between P and HZSM-5 and the nature of the acid sites thus induced are worthy of further clarification because they are closely related to the anti-coke deposition as well as the catalytic behavior of the catalyst.

Recently, we prepared a series of catalysts for C_4_-olefin cracking by impregnating the HZSM-5 with different amounts of phosphoric acid, followed by calcination in air at high temperature [93]. XRD patterns showed that the structure of zeolite framework was scarcely damaged after P modification. NH_3_-TPD and IR spectra indicated decreased acidity in the P-modified HZSM-5. The modification also resulted in dealumination of the HZSM-5 framework. The introduction of a suitable amount of phosphorus into HZSM-5 zeolite can improve its catalytic performance in the cracking of C_4_-olefins. Both coke deposition and dealumination were the crucial causes of HZSM-5 deactivation. It was found that P modification could enhance the catalyst’s anticoking ability in C_4_-olefin cracking raction, which is mainly due to the fact that P modification, had caused dealumination and thus eliminated part of the strong acid sites in HZSM-5. During the C_4_-olefin cracking reaction process the tetrahedral aluminum framework of the P-modified HZSM-5 was changed mainly to distorted tetrahedral aluminum or pentacoordinated aluminum species which still could act as stable active centers and hence, the P-modified HZSM-5 showed excellent stability. The dimerization-β-scission mechanism was a dominant process for C_4_-olefin cracking to produce propylene. HZSM-5 modified with an appropriate amount of P (1.5 wt%) was proven to be the optimal catalyst for C_4_-olefin cracking to produce propylene (lifetime exceeding 800 h on a single pass, and it could probably be applied to an industrial process).

New kind of acid sites created in phosphorus-modified HZSM-5 through treatment in 100% steam at 1,073 K show much better hydrothermal stability and significantly modified catalytic performance for C_4_-olefin cracking. Our investigation using D_2_/OH exchange and solid-state ^31^P- and ^27^Al-NMR measurements show that the new acid sites seem to be related to the phosphorus entering into the zeolitic position left by dealumination and stabilized by some extra-lattice aluminum [94]. The entry of phosphorus into the zeolitic framework protects the residual framework from further dealumination. The integrated effect causes the hydrothermally stable acid sites in P-modified HZSM-5 and enhances the catalytic performance for C_4_-olefin cracking after steam treatment at high temperature.

**Figure 2.**
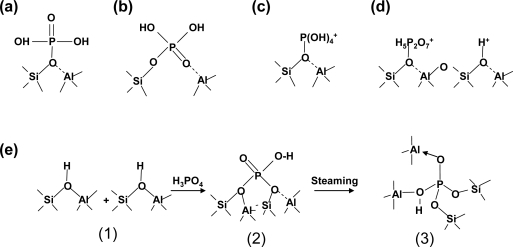
Models proposed for the interaction of phosphorus with the Brönsted acid sites of HZSM-5 prepared by impregnation with phosphorus modifiers and calcinations. **(a)** proposed by Kaeding *et al.* [95] and Vedrine *et al.* [96]; **(b)** proposed by Lercher *et al.* [97], **(c)** and **(d)** proposed by Corma *et al.* [98]; **(e)** proposed in this work [94].

At present, it is difficult to describe the exact structure of the new acids formed in P/HZSM-5 during steam treatment. As early as 1986, Lercher *et al.* [97] proposed a model to describe the interaction between the bridged hydroxyls of ZSM-5 and orthophosphoric acid. Corma *et al.* recently summarized the models appearing in the literature describing the interaction between HZSM-5 and phosphorous species for elucidation of the status of phosphorus species and hydroxyl groups [95–98,99]. One widely stated view is that the framework aluminum, or a part of the framework aluminum, is stabilized via the interaction with phosphorus species. This has been convincingly supported by ^27^Al-NMR measurements. However, these models cannot explain the changes in the number of OH groups in a series of P/HZSM-5 catalysts. Apparently, a model to account for the structure of the modified acid sites has not yet been proposed. To summarize the current results, Figure 2 shows the schematic mechanisms for the interaction of phosphorus with HZSM-5 (Figure 2e), along with other previously proposed models (Figure 2a, 2b, 2c, and 2d).

It seems that these four models cannot account for the change in the number of OH groups. According to the model we proposed in Figure 2e, two zeolitic hydroxyls condense with one phosphate molecule before the steam treatment but calcined in dry air, leading to a decrease in the number of OH groups on P modification [Figure 2e(2)], which was in agreement with the results of Corma *et al.* [98]. During the steam treatment (dealumination), some zeolitic OH groups, (SiO)_4_Al–H, were substituted by phosphorous hydroxyls (P–OH), stabilized by nonframework Al, which are hydrothermally stable [Figure 2e(3)]. More recently, Blasco *et al.* [98] proposed a new model concerning the interaction between orthophosphoric acid and bridged hydroxyls before steam treatment. They proposed that the phosphorous stabilized framework aluminum pairs (Figure 2c and 2d). Our current results support this proposal. However, the situation changed due to steam treatment at high temperatures. A new kind of OH group was formed. We believe that the phosphorus entered the framework by occupying the vacancies left by dealumination during steam treatment. The framework P stabilized by nonframework Al resulted in a new kind of acid site that is hydrothermally stable. The mechanism reported herein should be very important in zeolite applications for hydrocarbon reactions.

### 2.3. Metal Oxide Decorated Zeolite Composite Catalysts

Based on our previous research on phosphorus oxide modified zeolite composite catalysts for C_4_-olefin cracking, we investigated the introduction of tungsten into phosphorus-modified HZSM-5 (P/HZSM-5) [100]. As we know, among heterogeneous acid catalysts under investigation, the heteropoly acids (HPAs) are strong Brönsted acids. It is unlikely that H_3_PW_12_O_40_ (HPW) would be formed in the zeolite channels by the introduction of tungsten into phosphorus-modified HZSM-5, but some interaction between tungsten and phosphorus species should be expected by doping tungsten species to P/HZSM-5. Furthermore, it has been documented that supported tungsten oxides are good catalysts for isomerization of hydrocarbons, e.g., 1-butene, *n*-decane and C_7_+ paraffins [101–105]. Considering these properties of tungsten and the anticipated interaction between phosphorus and tungsten, we concluded that doping tungsten into P/HZSM-5 would further promote its catalytic performance for 1-butene cracking.

**Figure 3.**
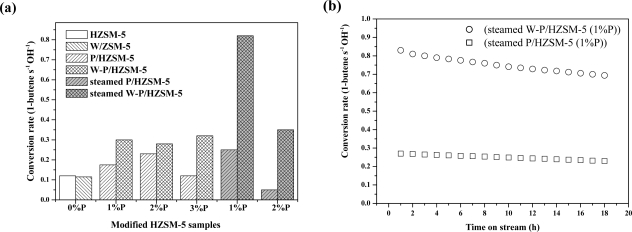
**(a)** List of the conversion rate of 1-butene over a series of catalysts at the same reaction conditions; **(b)** relative catalytic performance of steamed P/HZSM-5 (containing 1%P) and steamed W–P/HZSM-5 (containing 1%P) samples as a function of time on stream (temperature: 803 K; WHSV = 13 h^−1^; N_2_/1-butene = 3). Adapted from Reference [100].

As expected, we found a synergistic effect among tungsten, phosphorus and HZSM-5 in 1-butene cracking to propylene and ethylene, which was demonstrated by catalytic tests [100]. The conversion rates of 1-butene cracking over the catalysts were calculated relative to OH density (shown in Figure 3a). It is clear that the tungsten-doped P/HZSM-5 (W–P/HZSM-5) shows better catalytic performance than P/HZSM-5; and the highest specific conversion rate is observed on the steamed W–P/HZSM-5 with 1% P. In comparison with P/HZSM-5, the enhancement in catalytic performance of W–P/HZSM-5 surely reflects the contribution of the doped tungsten. The conversion rate of 1-butene over W/HZSM-5, however, is just slightly affected by the tungsten doping. Hence, a synergistic effect between the tungsten and phosphorous on the acid sites of zeolite can be infered. The structure similar to the HPW species might be formed in and out of zeolitic channels and is responsible for the enhanced conversion rate. The new acid sites for the interaction of P and W are also deduced by D_2_/OH exchange and NH_3_ adsorption microcalorimetry. After steam treatment at 1073 K for 4 h, the steamed W–P/HZSM-5 catalysts show more distinct cracking enhancement, as shown in Figure 3a. This result leads to the conclusion that the synergistic effect of P and W is very stable in steam at high temperature. Figure 3b shows the specific activity of 1-butene cracking over steamed P/HZSM-5 and steamed W–P/HZSM-5 with time on stream. It can be seen that the specific activity of steamed W–P/HZSM-5 sample is much higher than that of steamed P/HZSM-5 for 1-butene cracking on the basis of OH groups. A similar deactivation is shown but the higher activity even with low acid density in W–P/HZSM-5 catalyst is quite attractive. Besides, according to the selectivity to propylene, W–P/HZSM-5 showed higher propylene selectivity than P/HZSM-5.

The enhanced performance of the catalyst for 1-butene cracking to propylene and ethylene is related to the synergistic effect between the doped tungsten and phosphorous on the reaction network of the cracking process. The W–P/HZSM-5 is a promising catalyst for the cracking of 1-butene to propylene and ethylene.

## 3. Composite Zeolites by Co-Crystallization or Overgrowth

Composite zeolites, especially micro/microporous composites [106–111] and micro/mesoporous composites [112–116], have been researched because of their synergistic performance in catalytic reactions. Generally, a pure zeolite phase can be obtained only when the synthesis parameters are well controlled, while the variation in the zeolite synthesis conditions will lead to the formation of cocrystallized zeolites with different phases. They may be regarded as the physical mixture in many cases, and their structure and properties are neglected. Only a few examples of intergrowth zeolites from cocrystallization, such as the intergrowth of zeolites X/A [117], MFI/MEL [118], FAU/EMT [119], and STF/SFF [120] with the analogous basic structures, have been reported so far. In some cases, composite materials are preferred in the catalytic reactions because of the combination of different structures and better performance [121,122]. Imbert *et al.* observed that the isomerization and hydrogen-transfer reactions could be inhibited within the pore systems of ZSM-5/ZSM-11 intergrowth zeolites for the n-decane cracking [123]. Besides, another ZSM-5/ZSM-11 composite, a co-crystalline zeolite [106], showed a high activity, selectivity and stability for the alkylation reaction of benzene with the dilute ethylene in FCC off gas, and ZSM-5/ZSM-11 intermediate [124,125] effectively catalyzed the conversion of methanol and the alkylation of aromatics. ZSM-5/Y composites consisting of either the mechanical mixture of ZSM-5 and Y [126] or the *in situ* synthesized ZSM-5/Y composites [127] were reported to exhibit a notable synergy in the catalysis. Recently, more microporous/mesoporous composites such as ZSM-5/MCM-41, Beta/MCM-41, Y/MCM-41 and Ti-Beta/SBA-15 are seeding catalytic applications as they can combine the advantages of microporous materials with high activity and stability and of mesoporous materials with large pore size [128]. At the same time, composite zeolites with binary structure also lead to special properties which can improve its catalytic performance.

We carried out a series of investigations on MWW/FER composite zeolite [129]. It was found that MCM-22/ZSM-35 composites could be rapidly crystallized at 174 ^°^C with an optimal gel composition of SiO_2_/Al_2_O_3_ = 25, Na_2_O/SiO_2_ = 0.11, HMI/SiO_2_ = 0.35, and H_2_O/SiO_2_ = 45 (molar ratio), of which the weight ratio of ZSM-35 zeolite in the composite relied on the crystallization time. Besides, a novel route for synthesis of MCM-49/ZSM-35 composite zeolites with different compositions in a mixed amine system containing hexamethyleneimine (HMI) and cyclohexamine (CHA) has been developed [130]. The MCM-49/ZSM-35 composites with more content of ZSM-35 zeolite can be prepared under conditions of larger CHA/HMI molar ratio, higher Na_2_O/Al_2_O_3_ molar ratio and longer crystallization time. In the same system, both MCM-49 zeolite and ZSM-35 zeolite can also be successfully synthesized by adjusting the synthesis conditions.

One- and two-dimensional ^129^Xe-NMR spectroscopy is a powerful tool for probing the porosity and species distribution in porous materials which has been employed to study the porosity of cocrystallized MCM-49/ZSM-35 zeolites under a continuous flow of hyperpolarized xenon gas. It was found by variable temperature experiments that Xe atoms can be adsorbed in different domains of MCM-49/ZSM-35 cocrystallized zeolites and the mechanically mixed counterparts [131]. The exchange of Xe atoms in different types of pores is very fast at ambient temperature. Even at very low temperature two-dimensional exchange spectra (EXSY) show that Xe atoms still exchange between MCM-49 and ZSM-35 analogues in the co-crystallized zeolites much faster than in the mechanical mixture. This demonstrates that the MCM-49 and ZSM-35 analogues in co-crystallized zeolites may be stacked much closer than in the physical mixture, and some parts of intergrowth may be formed due to the partially similar basic structure of MCM-49 and ZSM-35.

In order to test the catalytic performance of the MCM-22/ZSM-35 composite catalysts, pulse reactions were carried out to study the initial activity of butene aromatization with the products formed directly detected by an on-line multi-channel MAS [129]. To our surprise, the synthesized MCM-22/ZSM-35 composite catalysts demonstrated relative higher aromatization ability than the pure phase of either MCM-22 or ZSM-35. Besides, with regard to their catalytic performance for the aromatization of olefins in LPG/FCC gasoline, the coexistence of MCM-22 and ZSM-35 in the composite (MCM-22/ZSM-35 = 45/55 wt/wt) was also observed to exert a notable synergistic effect on the aromatization ability for butene conversion and FCC gasoline updating, which is possibly due to the intergrowth of some MCM-22 and ZSM-35 layers [129].

**Figure 4.**
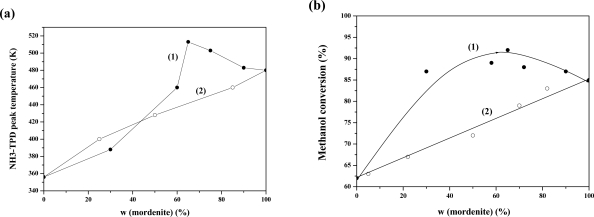
**(a)** Influence of mordenite phase content on the higher NH_3_-TPD peak temperature; **(b)** Influence of mordenite phase content on catalytic performance of BEA/MOR and mechanical mixture of zeolite beta and mordenite for methanol dehydration (1)- BEA/MOR, (2)-mechanical mixture. Adapted from Reference [133].

The second composite zeolite we investigated was BEA/MOR (a co-crystalline zeolite of beta and mordenite) zeolite composite which was synthesized using tetraethylammonium fluoride as composite template [132]. The crystallization process of the BEA/MOR composite zeolite had been systematically investigated by XRD, ICP, SEM, TGA, and nitrogen adsorption characterizations. It was found that the beta and mordenite phases did not appear simultaneously in the crystallization process when the crystallization was going on. The beta phase was the favored product at the beginning of crystallization, thereafter the mordernite phase emerged, and the BEA/MOR composite zeolite was gradually formed. The morphology of obtained particles changed a bit during the crystallization process, but the granularity distribution range broadened. Asymmetry of BEA/MOR composite zeolite particles led to uneven distribution of the mesopores. The acid properties of BEA/MOR samples were characterized by temperature programmed desorption of NH_3_ (NH_3_-TPD) [133]. There is a volcano-type relationship between the higher NH_3_-TPD peak temperature and the mordenite content of the BEA/MOR samples, and the NH_3_-TPD peak temperature reached the maximum at mordenite content of about 65% (Figure 4a).

In contrast, there is a linear relationship between the higher NH_3_-TPD peak temperature and the mordenite content of the mechanical mixtures of zeolite beta and mordenite. The NH_3_-TPD peak temperature represents the acid strength of the zeolite catalysts. The catalytic activity measurements of all the samples for methanol dehydration were carried out in a fixed bed reactor under atmospheric pressure, temperature of 180°C, and methanol space velocity of 2 h^−1^. Dimethyl ether and water were the only products for each catalyst. No coke formation and catalyst deactivation were observed in a 6 h reaction. There is a linear relationship between the methanol conversion and the mordenite content of the mechanical mixtures. The BEA/MOR samples with mordenite content higher than 40% showed higher catalytic activity than mordenite, and the BEA/MOR with 65% mordenite showed the highest methanol conversion (Figure 4b). The catalytic activity of BEA/MOR also exhibited a volcano-type behavior. Compared with the mechanical mixtures, the BEA/MOR samples displayed higher catalytic activity, which should be closely related to the acid strength of the zeolite catalysts.

**Figure 5.**
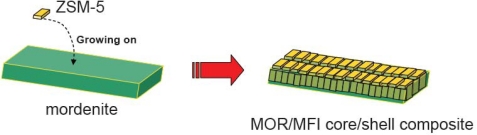
Growth of MOR/MFI core/shell composite. Reproduced with permission from Elsevier [134].

Another composite zeolite we investigated was a MOR/MFI core/shell composite [134]. It was successfully fabricated by the overgrowth method, through which mordenite cores were firstly pre-treated with an amine solution and then ZSM-5 shells overgrown on the surface of the mordenite crystallites (Figure 5). It was found that the first pre-treatment step was crucial for the fabrication of MOR/MFI overgrowth structures. As long as the core crystals comprise the elements required for the formation of the shell phases, continuous shell layers can be initiated and fabricated around the core crystals. Current methodology avoided the utilization of frangible silicalite-1 nanoseeds and costly starting materials. The flexible nature of current approach would allow the synthesis of other couples of core/shell zeolites despite the structural or chemical incompatibilities.

## 4. Hierarchical Porous Composite Catalysts

As we know, the physical properties of pores or pore channels of porous materials, such as the pore shape, pore size and pore structures, are closely related to the adsorption, diffusion and shape confinement of molecules in catalytic reactions, which will exert impact on activity, selectivity and life of the catalysts. So it is important to select, fabricate and adjust proper pore systems of the catalysts.

The zeolite-based hierarchical porous materials with two or more levels of porosity have potential applications in several industrial fields [135]. These materials have been proven to be promising catalysts that combine the advantages of shape selectivity with efficient mass transport [136]. The presence of mesopores in zeolite has improved theircatalytic activities and selectivities significantly as compared to conventional zeolite catalysts in a series of catalytic reactions, such as the alkylation of benzene with ethylene over ZSM-5[137], the cracking of lager molecule 1,3,5-triisopropylbenzene and cumene [138,139],the epoxidation of alkylene over TS-1 and TS-2 [140,141], isomerization of 1,2,4-trimethylbenzene, and esterification of benzylalcohol with hexanoic acid. Most importantly, some zeolites with mesoporosity have been used in a number of industrial processes including the cracking of heavy oil fraction over zeolite Y, the production of cumene, hydro-isomerization of alkanes over mordenite, and the synthesis of fine chemicals over Y, ZSM-5, and beta [142]. The enhanced catalytic activities are due to the reduction of diffusion path and easier access to the active site as a result of the introduction of mesopores into the zeolite framework [143,144].

Various attempts have been undertaken to synthesize composite materials which combine the structures of both zeolites and mesoporous molecular sieves, such as the recrystallization of the walls of SBA and MCM materials in the presence of molecular templates [145], the preparation of zeolite coated mesoporous aluminosilicates by using diluted clear solutions containing primary zeolite units [146], and the assembly of zeolite nanoblocks into ordered mesoporous materials [147]. The introduction of mesoporosity into zeolite materials by different approaches is another method for the synthesis of mesoporous zeolite materials [148]. The obtained mesoporous zeolites preserve the crystalline nature of the zeolite materials, and the mesoporosity in zeolite can increase accessibility to the internal surface. The post-synthesis technique is a traditional method for the generation of mesopores within zeolite crystals. Upon steaming [149], acid or base leaching [150,151] and chemical treatment, the aluminum or silicon atoms can be selectively extracted from the framework, resulting in the formation of mesopores in zeolite framework. An alternative route for the preparation of mesoporous zeolite is to introduce mesopores into zeolite crystal during the zeolite crystallization. Besides a structure-directing agent for templating the zeolite structure, an easily removable pore-filling additive in the dimension of nanoscale is also often used to create mesopores in zeolite crystal. Zeolite crystallized around hard templates such as carbon black [152], carbon nanotube [153], carbon aerogel [154], mesoporous carbon [155], wood cell [156], and organic gel [157]. Moreover, a few water soluble polymers with strong interaction with silica can be incorporated into silicate crystal during the crystallization, and then they are removed to generate moso-porosity [158,159]. Recently, Choi’s group has developed a direct synthesis route to mesoporous zeolites with tunable mesoporous structure by using the amphiphilic organosilane assembled supramolecule as a mesopore-directing agent [160]. The methods mentioned above make the synthesis of mesoporous zeolite possible, constructing a new family of zeolite-based materials with multilevel of pore size distribution.

**Figure 6.**
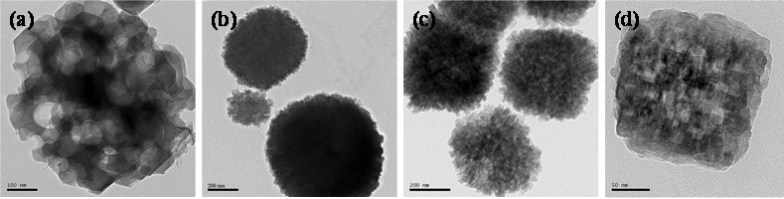
Hierarchical porous zeolites: **(a)** mesoporous silicalite-1 synthesized with nano CaCO_3_ templates. Adapted from Reference [161]; **(b)** mesoporous ZSM-5 synthesized with starch templates. Adapted from Reference [162]; **(c)** mesoporous ZSM-11 synthesized with polyvinyl butyral gel templates. Adapted from Reference [163]; **(d)** mesoporous Beta zeolite synthesized with polyvinyl butyral gel templates. Adapted from Reference [163].

We have successfully synthesized several kinds of hierarchical porous zeolites (silicalite-1 [161], ZSM-5 [162], ZSM-11 [163], Beta [163]) by using nanosized CaCO_3_, starch and polyvinyl butyral gel as templates (Figure 6). Upon the direct hydrothermal crystallization of templates/silica composite, the silica was transformed into zeolite, whereas the templates were incorporated in the zeolite crystals. Removal of the templates inside the zeolite composite yielded the opening of micropores and mesopores in zeolites, respectively (Figure 6). The characterization techniques confirmed that the obtained porous materials had well-defined microporosity and irregular mesoporosity.

The porous structures of mesopore ZSM-5 prepared by starch templates have been studied by continuous-flow laser-hyperpolarized ^129^Xe-NMR [164]. Different adsorption domains of Xe in these hierarchical porous zeolites can be directly observed by variable-temperature experiments. The exchange of Xe atoms in different types of pore environments is very fast at ambient temperatures. Combining with nitrogen physisorption, we found that the Si/Al ratios of resultant samples have great influence on the mesopore size distribution and mesopore volume in meso-ZSM-5. Two dimensional exchange NMR spectroscopy (EXSY) has been used for the first time on such hierarchical zeolites, and the result showed that Xe atoms still undergo faster exchange between different types of pores in the meso-ZSM-5 than in the mechanically mixed ZSM-5/silica counterparts even at very low temperature. The result demonstrated that microporous and mesoporous domains in the meso-ZSM-5 may stay much closer and have better connectivity than in the conventional physical mixture, which facilitate the Xe atoms diffusion and exchange.

The cracking of 1,2,4-trimethylbenzene and 1,3,5-trimethylbenzene had been detected over the conventional ZSM-11 and mesoporous ZSM-11, respectively [163]. The conversion of trimethylbenzene is much higher in the mesoporous ZSM-11 than in the conventional ZSM-11. Apparently, the presence of mesopores in the ZSM-11 crystals makes for short diffusion paths within the zeolite particles, which facilitates reactant accessibility to the active sites on the interior surfaces. Experimental results also show that there are some differences in the product distribution between the mesoporous zeolite and conventional zeolite. In contrast to conventional ZSM-11catalysts, the mesoporous zeolite can stimulate the formation of bulky molecules, namely, C_10_ aromatics, in the trimethylbenzene cracking reaction. At the same temperature, the amount of C_10_ aromatics is larger in the mesoporous ZSM-11 than in the conventional ZSM-11, which may be a result of the higher conversion of trimethylbenzene in mesoporous ZSM-11. We also compare the product distribution when the conversions reach the same level at different temperature. For example, the conversion of 1,2,4-trimethylbenzene on the mesoporous ZSM-11 at 350°C (33.57%) is nearly equal to that on conventional ZSM-11 at 390°C (32.35%), but there are more C_10_ aromatics with the former zeolite (2.14%) than with the latter zeolite (1.65%). Also, in the 1,2,4-trimethylbenzene cracking reaction, the conversion of 1,2,4-trimethylbenzene on the two ZSM-11 zeolites reaches the same level at 410 °C (52.78% for the mesoporous ZSM-11 and 50.10% for the conventional ZSM-11), but the amount of C_10_ aromatics on mesoporous ZSM-11 (5.00%) is about twice as much as that seen on conventional ZSM-11 (2.91%). Meanwhile, there is a similar tendency in the cracking of 1,3,5-trimethylbenzene over conventional and mesoporous ZSM-11, and the large molecules (C_10_ aromatics) are also apt to be produced on the mesoporous ZSM-11. Zeolites with large pores are generally beneficial for the formation of the bulky molecules, whereas small-pore zeolites favor the production of small molecules. The presence of mesopores within ZSM-11 can accommodate the guest molecules that are larger than its intrinsic 10-membered pores. Therefore, the mesoporous ZSM-11 promotes the formation of the large molecules.

**Figure 7.**
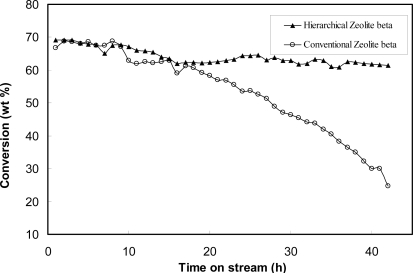
Deactivation behavior of mesoporous zeolite beta and conventional zeolite beta for the cracking of 1,2,4-trimethylbenzene at 350 °C. Adapted from Reference [163].

Zeolite beta has open-framework structure of three-dimensional 12-membered ring channels, and its pore size is larger than that of ZSM-11, so the diffusion of trimethylbenzene becomes easier in zeolite beta than in ZSM-11. It is found that the catalytic activity of the mesoporous zeolite beta is not much higher than that of mesoporous ZSM-11. Especially at 350 °C, the conversion of 1,2,4-trimethylbenzene over the mesoporous zeolite beta and conventional zeolite beta are of the same level. Mesoporous ZSM-11 and conventional zeolite beta suffered from deactivation at 350 °C for a long time, while the mesoporous zeolite beta shows good resistance to deactivation at this temperature. As shown in Figure 7, two zeolite beta samples display similar activity during the initial 15 h. However, with the passage of time, the two zeolite samples exhibit remarkably different behaviors. That is, catalytic activity of the conventional zeolite beta decreases very rapidly with time, whereas no significant deactivation is observed in the case of the mesoporous zeolite beta. For mesoporous zeolite beta, the conversion remains at a stable level (about 66%) during 14–42 h, whereas for the conventional zeolite beta, the conversion gradually drops to a low level, from 70 to 24%, as shown in the Figure 7, indicating that this catalyst deactivates quickly with the time on stream. The catalyst recovered after the reaction turns into a black material, which is associated with coke formation during the reaction. As compared with the conventional zeolite beta, the mesoporous zeolite beta can suppress the deactivation that is attributed to the easy transportation of molecules with large dimension through mesopores. The deactivation of the zeolite catalyst is a very complex process, but it is believed that the coke formation through the successive reactions of reactant and product molecules is the main reason for the deactivation of microporous catalysts. A large number of the mesopores in mesoporous zeolite beta would accelerate molecule diffusion out of the zeolite crystal; hence, their successive reaction in the active sites would be suppressed dramatically. Therefore, the micropore blockage by coke would be alleviated significantly because of the facile diffusion of large molecules in the hierarchical pore system. The introduction of mesopore into conventional zeolite may be a valuable method to inhibit the coke formation.

## 5. Host-Guest Porous Composite Catalysts

Chirality is one of the attributes of Nature, which is involved with many of the behaviors observed and some basic natural laws. In Nature, a molecule's chirality is often as important as its chemical makeup. Most biomolecules – proteins, hormones, nutrients, sugars, fats, and many others – are chiral. Besides, it is also critical in the biochemistry of natural products and drugs, so basic research on chiral compounds is another important research focus. Chiral compounds can be obtained by many methods among which the chiral catalytic syntheses are always considered to be the most economical and rational. Up to now, most of the chiral catalysts are homogeneous, which is good for catalytic efficiency but hard for recovery. The recovery of homogeneous catalyst and its purification are usually obstacles to the practical application of these catalysts. To solve the problem, heterogenization of homogeneous chiral catalysts are been developed [165–174]. In most cases, however, the immobilized catalysts exhibit lower activity and enantioselectivity and they are located in nanopores [175]. The development of an efficient method for the heterogenization of chiral catalysts still remains a challenging objective.

The entrapment of homogeneous catalysts within the porous matrix, compared with other immobilization methods such as covalent-bonding and electronic interaction, is highly desirable because in principle the trapped homogeneous catalyst should retain the properties of its free form. Mesoporous silicas with an ordered pore arrangement, a high surface area and a rigid framework are ideal porous materials to trap catalysts. Balkus and co-workers attempted to encapsulate an enzyme within the channel of MCM-41 [176]. Compared with enzymes, most homogeneous catalysts are much smaller in size. Therefore, it is more difficult to trap the homogeneous catalyst within the mesoporous silica while still keeping the catalyst as free as in homogeneous medium. As far as we know, a successful entrapment of a small metal complex, especially a chiral metal complex, within mesoporous silica has been rarely reported so far.

**Figure 8.**
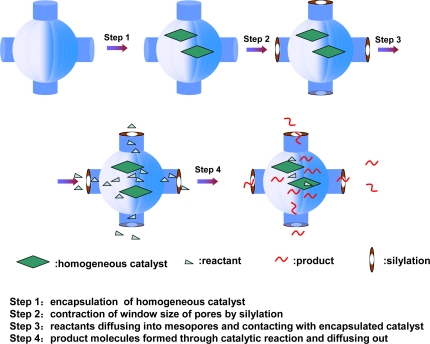
Schematic description of entrapping homogeneous catalyst within the cage of mesoporous silicas. Reproduced with permission from Royal Society [175].

We have developed a novel host-guest method to prepare heterogeneous asymmetric composite catalysts through entrapment of metal complexes within the cages of mesoporous silica [177], as illustrated in Figure 8. A preformed homogeneous catalyst is first introduced into the cage-like pore of a mesoporous material (e.g., SBA-16 with tunable small pore entrance and large cage size [178,179]) by impregnation or adsorption. The pore entrance size is finely tailored by a silylation method, according to the molecular size of the catalyst, reactants and products. Thereby the metal complexes can be confined in the mesoporous cages. Reactants and products (usually smaller than the size of the pore entrance) are allowed to freely diffuse through the entrance to the mesoporous cage, in which the entrapped catalysts can speed up the reaction with high activity in a similar way to that in the actual homogeneous catalysis process. The advantage of this strategy over other immobilization methods is that the identity of a homogeneous catalyst can be largely kept because the trapped catalyst is almost intact.

By this host-guest method, a novel composite catalyst, Co(Salen)/SBA-16-C8, was prepared by the encapsulaton of Co(Salen) in SBA-16 with silylation [177]. The fresh Co(Salen)/SBA-16-C8 with silylation gives a comparable diol yield and ee (43% yield and 91% ee) as its homogeneous counterpart for hydrolysis reaction of epoxychloropropane. For comparison, the catalyst without silylation was also prepared. It was found that the catalyst without silylation is nearly inactive for this hydrolysis reaction, indicating that most of the adsorbed Co(Salen) was washed away from SBA-16 during the catalyst preparation because of the large pore entrance size. This result indicates that silylation is indispensable for confining metal complexes inside the mesoporous cages. In addition, the catalyst can be convconstant even after the fifth use. The Co(Salen)/SBA-16-C8 can even be run for twelve times (33% yield and 88% ee). During the catalyst recycle process, a longer reaction time was needed to complete the reaction compared with the fresh catalyst. This is partly due to the loss of solid catalyst during the course of recovery (for the 12th run, the catalyst weight is *ca.* 65% of the original one). The filtrate does not show any activity for the ring-opening reaction. This confirms that the conversion is contributed by the catalyst trapped in the cage of the mesoporous material. In addition, the Co(Salen)/SBA-16-C8 catalyst was also used to catalyze the asymmetric ring-opening of propylene oxide. No obvious loss of activity and ee was observed even when Co(Salen)/SBA-16-C8 was run for the thirteenth time. After the thirteen cycles of reaction, the Co content in Co(Salen)/SBA-16-C8 was 87% of that seen in the fresh one.

**Figure 9.**
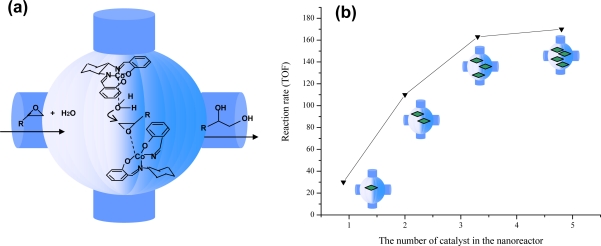
**(a)** Hydrolytic kinetic resolution of epoxides on [Co(salen)] complexes confined in the nanocages of SBA-16; **(b)** The catalytic activity of [Co(salen)]/SBA-16 as a function of [Co (salen)] number in each cage. Reproduced with permission from Wiley-VCH [180].

To study the effect of loading amount of [Co(salen)] on catalytic performance, we prepared four solid catalysts with Co contents of 0.055, 0.087, 0.157, and 0.225 wt%, corresponding to 1.2, 1.9, 3.4, and 4.9 [Co(salen)] complexes, respectively, accommodated in each nanocage of SBA-16[180]. It was surprising to find that the [Co(salen)]/SBA-16 catalysts with two or more than two [Co(salen)] complexes in each cage show a significantly enhanced cooperative activation effect and exhibit much higher activity than the homogeneous [Co(salen)] catalyst for the hydrolytic kinetic resolution (HKR) of epoxides (Figure 9a). The catalytic activity gradually increases as the number of [Co(salen)] units in each cage increases, and then reaches a plateau when the number reaches five (Figure 9b). The increase in the activity and enantioselectivity with an increase in the number of [Co(salen)] complexes per cage obviously indicates that the cooperative activation effect of [Co(salen)] complexes in the nanocages can be strengthed owing to the crowded situation of the cobalt complexes in the nanocages. The appropriate proximity and the free movement of [Co(salen)] in the confined space offer the possibility that H_2_O activated by one [Co(salen)] complex can attack the epoxide activated by another [Co(salen)] complex, and then facially produce the diol with high activity. This reasoning is corroborated by DFT calculations.

Besides, another new heterogeneous composite catalyst, Ru(TsDPEN)/SBA-16-2Ph, was prepared by the encapsulation of Ru(TsDPEN) in the mesoporous cage of SBA-16 using diphenyldichlorosilane as silylation agent [177]. TsDPEN is among the most efficient catalysts for the asymmetric transfer hydrogenation of prochiral ketones [181,182]. The heterogeneous catalyst Ru(TsDPEN)/SBA-16-2Ph exhibits comparable enantioselectivity with the homogeneous counterpart. The recycle reaction test shows that similar conversion and ee value are obtained for the first three runs. When the catalyst was run for the sixth time, the conversion could still reach 99%. Longer reaction time was needed to complete the reaction, partly due to the loss of solid catalyst during the course of recovery. The activity is relatively lower than that of the homogeneous catalyst because of the diffusion limitation. Moreover, Ru(TsDPEN)/SBA-16 can catalyze different ketones to corresponding chiral alcohols with high activity and enantioselectivity.

In conclusion, the chiral metal complex catalysts can be trapped in the cage of mesoporous materials like SBA-16 by modifying the entrance pore size of the cage using silylation. The entrapped chiral catalyst can show catalytic performance comparable to that of a homogeneous catalysis process and can be easily recycled without significant loss of performance. This strategy could be generally applicable for various chemical transformations and nano-reactor design.

## 6. Inorganic and Organic Mesoporous Composite Catalysts with Sulfonic Acid Functionality

The diverse applications of mesoporous materials in the fields of separation, chromatography, large molecular release systems, and catalysis have stimulated the search for materials with new structures and framework compositions [183–185]. The recent discovery of the periodic mesoporous organosilicas (PMOs) derived from a number of bridged organosilane precursors represented by the general formula (R’O)_3_Si-R-Si(R’O)_3_, where an organic group is an integral part of the mesoporous network, renewed interest in the applications of mesoporous materials [186,187]. Depending on the organic species in the network, the chemical and physical properties of PMOs can be tuned for desirable end uses. Recent advances in this area have demonstrated that PMOs can be synthesized with improved hydrothermal and mechanical stability compared to the conventional MCM-41 type mesoporous silicas [188]. The approaches generally used to expand the possibilities for tailoring the physical/chemical properties of hybrid silica are the combination of organosilane precursors with organic groups either bridging in the framework or dangling into the channel pore. Such bifunctionalized periodic mesoporous organosilicas (BPMOs) have already been reported in the literature [189,190]. Furthermore, due to the limited choice and unavailability of commercial bridged organosilane precursors, it is worth exploring the possible combinations of existing organosilane precursors with alkoxysilanes for the synthesis of PMOs and to study their structural relationship properties. In addition, the incorporation of transition metals into the framework of mesoporous organosilicas was also adopted in epoxidation and ammoximation reactions [192,193]. In recent years, sulfonic acid functionalized mesoporous materials were reported to be efficient catalysts for acid catalyzed reactions [194]. For practical applications, the hydrothermal stability of the materials is very important because most of the industrially important acid catalyzed reactions such as esterification, hydration, and condensation always involve water. The previously reported sulfonic acid modified mesoporous materials are hydrothermally unstable, and the mesostructures tend to collapse when they are subjected to prolonged water contact.

Incorporating the sulfonic acid functionality into mesoporous organosilicas improves the hydrothermal stability of the catalysts because the bridged organic groups in the mesoporous network make the materials hydrophobic. In this regard, we studied the synthesis of hydrophobic sulfonic acid functionalized benzene-, biphenylene-, and ethane-bridged mesoporous organosilicas for possible use as solid acid catalysts [195,196].

**Figure 10.**
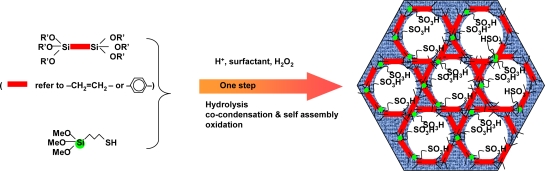
Direct synthesis of sulfonic acid-functionalized mesoporous organosilicas. Reproduced with permission from Elsevier [197].

We used two methods to prepare such sulfonic acid functionalized mesoporous organosilica composites. One is direct synthesis [197] in which ethane- or benzene-bridged organosilane or tetramethoxysilane (TMOS) was co-condensed with 3-mercaptopropyltrimethoxysilane [MPTMS: (MeO)_3_Si–CH_2_CH_2_CH_2_SH] in the presence of H_2_O_2_ using a nonionic oligomeric polymer surfactant (Brij-76 or P123) in an acidic medium. During the condensation process, thiol (-SH) group was oxidized *in situ* to a sulfonic acid (-SO_3_H) by hydrogen peroxide (30 wt % H_2_O_2_) (see Figure 10).

**Figure 11.**
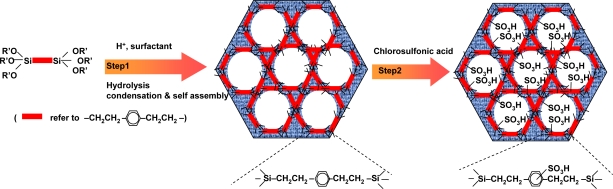
Schematic illustration of sulfonation process of the two step method. Reproduced with permission from Elsevier [198].

The other method is a two-step route [198] in which periodic mesoporous organosilicas (PMOs) containing different fraction of 1,4-diethylenebenzene groups in the framework were first synthesized by co-condensation of 1,4-bis(trimethoxysilylethyl)benzene with tetramethoxysilane under acidic conditions using a triblock copolymer as a structural directing agent. Then the sulfonic acid sites were incorporated in the mesoporous framework by post-synthetic framework modification of 1,4-diethylenebenzene by sulfonation with chlorosulfonic acid (see Figure 11). Through elemental analysis, it can be estimated that about 40% of the organic groups within the mesoporous framework are available for further chemical reaction.

The catalytic activity of these sulfonic acid functionalized mesoporous organosilicas was tested in the liquid-phase condensation of phenol with acetone to form bisphenol A, which is a key raw material for the synthesis of resins and polymers. In the commercial process, ion-exchange resins are often used as catalysts, but they display poor thermal stability. The search for effective solid acids with high thermal stability continues, and it has been found that the sulfonic acid-functionalized mesoporous organosilicas synthesized in this work meet all the requirements well [197]. The result is also supported by others [199,200] who claim that PMOs exhibit very good mechanical and hydrothermal stability.

In addition, the −SO_3_H functionalized mesoporous materials were found to be effective catalysts in the esterification of ethanol with acetic acid or aliphatic acid, and the results demonstrate that the ethane groups incorporated in the mesoporous framework have a positive influence on the catalytic behavior of the materials[198].

## 7. Polymer/CNT Composite Catalysts

Since the discovery of carbon nanotubes (CNTs) in 1991 by Iijima [201] and due to their unique physical properties [202,203], many researchers have tried to fabricate advanced CNT composite materials that exhibit one or more of these remarkable physical and mechanical properties [204–206]. One of the topics of current interest is to use CNTs as a filler in polymer composite systems to obtain ultra-light structural materials with enhanced mechanical, electrical, thermal and optical characteristics [207–213]. The prospect of obtaining advanced nanocomposites with multifunctional features, e.g., materials used for structures and electrical conductors, has attracted great efforts from researchers in both academia and industry [214–217].

Monoethylene glycol (MEG) is a necessary monomer for the production of polyester PET (polyethylene terephthalate), which is produced by hydration of ethylene oxide (EO). Non-catalytic and catalytic hydrations of EO are two well-known approaches to prepare MEG. Numerous catalysts, including anion exchange resins, quaternary phosphonium halides, polymeric organosilane ammonium salts and macrocyclic chelating compounds, have been used for the catalytic hydration of EO, and most of them use catalysts immobilized on ion exchange resin (IER) [218–220]. However, catalyst stability is a major challenge because the resins swell under the reaction conditions [221,222], and therefore there is an urgent demand for catalysts with high thermal stability while maintaining high selectivity and conversion. According to the papers published so far on heterogeneous catalytic hydration of ethylene oxide, polymeric ion exchange resins (IER), both strong acid and strong basic type, are the most effective catalysts. The acidic hydration process is accompanied with side reactions. On the other hand, alkaline hydration, with less byproducts, can increase the MEG selectivity, and is thus of greater research interest, but the intrinsic swelling problem of IER as the hydration proceeds restricts its application in the industrialization of catalytic hydration of ethylene oxide. Measures such as increasing the cross-linking density have been used to solve the swelling problem to some extent at the expense of reducing exchange capacity and the number of active sites of the resin.

**Figure 12.**
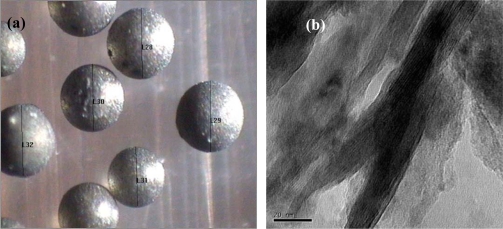
**(a)** A micrograph of catalyst beads (the scale bar is 1 millimeter); **(b)** TEM image of resin/CNT composite, showing the carbon nanotubes stuck within the resin matrix. Adapted from Reference [223].

We have developed an ion exchange resin/CNT composite (PS-DVB/CNT) to handle the problem of swelling as mentioned above [223]. This resin/CNT composite was prepared via *in-situ* suspension polymerization of styrene, divinylbenzene and multi-walled carbon nanotubes (MWNTs), followed by chloromethylation and amination. The pristine MWNTs, which acted as potential cross-linking agent, were dispersed well in the cross-linking network of PS-DVB polymer (Figure 12). The growing polymer chains, initiated by thermal decomposition of AIBN and subsequent polymerization, have tendency to add the double bonds on the surface of MWNTs, besides its tendency to add to the double bond of DVB, therefore we can readily functionalize the MWNTs through the “grafting-onto” mechanism. Taking the amount of double bonds into consideration, the MWNTs, together with DVB, might play the role of potential cross-linking agent. In our case, the content of MWNTs in the final resin composite reached 4% and the cross-linking density was 8%, equal to the industrialized non-functionalized ion exchange resin. It was found that the anti-swelling capability and thermal stability of resin/CNT composite are improved significantly after the addition of MWNTs while keeping the total number of active reaction sites, and the stability and the conductivity of the catalyst increased as well. It was also found that the glass transition temperature (*T_g_*) increased from 125 °C to 132 °C when MWNTs were incorporated in the cross-linking network of PS-DVB. The swelling extent of the composite could be reduced to almost half of that of PS-DVB, which indicated that the anti-swelling capability is improved after incorporation of carbon nanotubes into the IER. Most importantly, the enhanced thermal stability of the polymer composite facilitates carrying out the reactions at higher temperature (100 °C), which can help to reduce the water/feedstock ratio from 20–25 to below 12, and thus greatly reduces the energy consumption for the whole process. The obtained polymer/MWNTs composite performed very well in the experimental catalytic hydration of ethylene oxide, in which there was no obvious decline in conversion and selectivity after 2,200 h. The remarkable stability should be ascribed to the incorporation of MWNTs in the cross-linking network and the ability to keep the active site loss at low level.

The application of the resin/CNT composite catalyst in the hydration of ethylene oxide is a typical example in which the addition of CNT can enhance thermostability and anti-swelling capability. The function of CNT is just like steel bar for concrete reinforcement. This idea for the polymer/CNT composite catalyst may be extended to develop other composite catalysts for other industrial reactions.

## 8. Conclusions and Outlook

In summary, we have presented an overview of the recent development of some composite catalysts based on porous materials for various reactions and processes of interest to the chemical industry. It can be seen that composite catalysts are helpful for achieving high performances. The main conclusions and functions of these composite catalysts are summarized as follows:

✧ Regarding amorphous oxide modified zeolite composite catalysts, we have studied amorphous silica decorated zeolite composite catalysts, phosphorus oxide modified zeolite composite catalysts and metal oxide modified zeolite composite catalysts, among which the former are used for selective toluene disproportionation, and the latter two are used for C_4_-olefin cracking to produce propylene. The amorphous silica deposition by SiO_2_-CLD method is to eliminate the external acidic sites of zeolite and reduce the entrance size of zeolite, which are crucial to the high selectivity of *para*-xylene. Because the phosphorus oxide species stabilized by nonframework Al result in a new kind of acid site that is hydrothermally stable, it is a good method for enhancing the anti-coking ability and hydrothermal stability of zeolite composite catalysts. In addition, synergistic catalytic effects have been found by introducing tungsten into phosphorus-modified HZSM-5.
✧ Composite zeolites, such as MWW/FER, BEA/MOR and MOR/MFI were synthesized by co-crystallization or overgrowth. It is found that these composite zeolites are somewhat different from mechanical mixtures of the individual zeolites. Characterizations demonstrate that the composite zeolites may be stacked much closer than in the mechanical mixtures, and intergrowth areas may be formed. Compared with mechanical mixtures of zeolites, composite zeolites have higher acid strength and higher activity for acid reactions (aromatization of olefins and methanol dehydration), which may be due to their unique composite structures.
✧ As far as hierarchical porous catalysts are concerned, we have tried combine micropores, mesopores and macropores in one catalytic material. Hierarchical porous zeolites, such as silicalite-1, ZSM-5, ZSM-11, Beta were synthesized by using nanosized CaCO_3_, starch and polyvinyl butyral gel as templates for the generation of mesopores and macropores. It was found that these hierarchical pores were connective with each other which could facilitate the diffusion of molecules. When studying the process of C_9_ aromatics cracking, more C_10_ bulky molecules were favorably generated on hierarchical porous zeolites than inside conventional zeolites. Besides, the hierarchical porous zeolites show much lower deactivation rate than conventional zeolites, which could be attributed to the easy transport of molecules with large dimensions through mesopores and macropores.
✧ As for host-guest porous composites, chiral metal complex catalysts can be trapped in the cage of mesoporous materials like SBA-16 by modifying the entrance pore size of the cage using silylation. The entrapped chiral catalyst can be easily recycled without significant loss of catalytic performance and shows catalytic performance comparable to that in a homogeneous catalysis process. The increase in the activity and enantioselectivity with an increase in the number of [Co(salen)] complexes per cage obviously indicates that the cooperative activation effect of [Co(salen)] complexes in the nanocages can be strengthened owing to the crowded situation of the cobalt complexes in the nanocages.
✧ In the field of inorganic and organic mesoporous composite catalysts, we have studied meso-porous organosilica composites with sulfonic acid functionalities. Two methods were applied in preparing such composites: one is a direct synthesis method in which a thiol (-SH) group was oxidized *in situ* to a sulfonic acid (-SO_3_H) by hydrogen peroxide; the other is a two-step route in which periodic mesoporous organosilicas containing different fractions of 1,4-diethylene-benzene groups in the framework were synthesized first, and then the sulfonic acid sites were generated by framework sulfonation of 1,4-diethylenebenzene with chlorosulfonic acid. It has been found that these sulfonic acid-functionalized mesoporous organosilicas show high catalytic activity and stability in the liquid-phase condensation of phenol with acetone to form bisphenol A and esterification of ethanol with acetic or other aliphatic acids.
✧ As swelling of polymeric ion exchange resins has been an obstacle to their application as catalysts in the catalytic hydration of ethylene oxide, we prepared resin/CNT composite catalysts to overcome this problem. The addition of CNT into a resin can greatly enhance its thermostability and anti-swelling capability during the reaction of hydration of ethylene oxide. The remarkable stability observed should be ascribed to the incorporation of MWNTs in the cross-linking network and the ability to keep the active site loss at low level.

Zeolite catalysts have been industrialized for more than 50 years. Along with the pressing requirements for energy saving and environmental protection, these catalysts are still under intense study. As can be seen, the most recent advances concerning composite catalysts made from porous materials mainly involve combining the advantages of composite components based on a good understanding of the chemical reactions, so that composite catalysts are good candidates for achieving high performances features including high activity, remarkable selectivity and good resistance to deactivation. It is, and will still be, a challenging subject to design composite catalysts based on porous materials rationally and successfully apply them in the chemical industry.
